# Brain white matter integrity and association with age at onset in pediatric obsessive-compulsive disorder

**DOI:** 10.1186/s13587-014-0013-6

**Published:** 2014-12-18

**Authors:** Isabelle M Rosso, Elizabeth A Olson, Jennifer C Britton, S Evelyn Stewart, George Papadimitriou, William DS Killgore, Nikos Makris, Sabine Wilhelm, Michael A Jenike, Scott L Rauch

**Affiliations:** Center for Depression, Anxiety and Stress Research, McLean Hospital, 115 Mill Street, mailstop 334, Belmont, MA 02478 USA; Department of Psychiatry, Harvard Medical School, Boston, MA USA; Department of Psychology, University of Miami, 5665 Ponce de Leon Blvd, Flipse Building, Coral Gables, Miami, FL 33146 USA; British Columbia Mental Health and Addictions Research Institute, University of British Columbia, Vancouver, BC Canada; Center for Morphometric Analysis, Massachusetts General Hospital, Bldg 149, 13th Street, Charlestown, MA 02129 USA; Department of Neurology, Harvard Medical School, Boston, MA USA; Department of Radiology, Harvard Medical School, Boston, MA USA; Department of Psychiatry, Massachusetts General Hospital, Simches Research Building, 185 Cambridge Street, Suite 2282, Boston, MA 02114 USA; UBC Psychiatry A3-118, 938 West 28th Ave, Vancouver, BC V5Z 4H4 Canada; Department of Psychiatry, University of Arizona, Tucson, AZ USA

**Keywords:** Obsessive-compulsive disorder, Childhood, Diffusion tensor imaging, Corpus callosum, Thalamus, Age at onset

## Abstract

**Background:**

Obsessive-compulsive disorder (OCD) is a common and debilitating neuropsychiatric illness thought to involve abnormal connectivity of widespread brain networks, including frontal-striatal-thalamic circuits. At least half of OCD cases arise in childhood and their underlying neuropathology may differ at least in part from that of adult-onset OCD. Yet, only a few studies have examined brain white matter (WM) integrity in childhood-onset OCD using diffusion tensor imaging (DTI), and none have examined potential associations with age at onset.

**Results:**

In this study, 17 youth with OCD and 19 healthy control subjects, ages 10 to 19 years, underwent DTI on a 3T Siemens scanner. DSM-IV diagnoses were established with standardized interviews, and OCD symptom severity was evaluated using the Children’s Yale-Brown Obsessive-Compulsive Scale (CY-BOCS). Voxel-wise analyses were conducted on data processed with tract-based spatial statistics (TBSS) to derive measures of fractional anisotropy (FA), axial diffusivity (AD), radial diffusivity (RD), and mean diffusivity (MD). OCD patients had significantly lower FA in seven WM clusters, with over 80% of significant voxels in bilateral frontal cortex and corpus callosum (CC). There were no regions of significantly higher FA in patients compared with controls. Patients also had significantly higher RD in right frontal cortex and right body of the CC. Earlier age at onset of OCD correlated significantly with lower FA in the right thalamus and with higher RD in the right CC. FA and RD were not significantly associated with symptom severity.

**Conclusions:**

These findings point to compromised WM integrity and reduced myelination in some brain regions of children with OCD, particularly the CC and fiber tracts that connect the frontal lobes to widespread cortical and subcortical targets. They also suggest that age at onset may be a moderator of some of the WM changes in pediatric OCD.

## Background

Obsessive-compulsive disorder (OCD) is a common neuropsychiatric illness characterized by repetitive thoughts and behaviors that are unwanted, distressing, and disabling (DSM-IV; [1]). Onset occurs in childhood for more than half of OCD patients, with about 40% of pediatric cases achieving remission and the remaining persisting into adulthood [2,3]. Epidemiological studies show that pediatric OCD has typical onset between 7 and 13 years [2] and that it differs from its adult-onset counterpart in a number of ways, including male preponderance, high familial loading, and more frequent comorbidity with developmental disorders [2,4]. Based on these correlates, it has been speculated that pediatric OCD may be a developmental subtype that is discontinuous from adult OCD, or a developmentally moderated expression of etiologic processes that are shared with the adult clinical phenotype [5,6].

Neuroimaging research has provided converging evidence that OCD symptoms arise from alterations in widespread brain networks. Abnormalities in frontal-striatal-thalamo-cortical loops are central to prevailing conceptual models of the disorder [7,8]. Pathological obsessions and compulsions are proposed to involve insufficient inhibitory control of striatal and thalamic nuclei by prefrontal cortical regions, particularly the anterior cingulate and lateral orbitofrontal cortices [9], and neuroimaging findings of altered regional brain volumes and function largely support these models (e.g., [10,11]). In addition, there is growing evidence implicating brain regions outside of the frontal-subcortical loops, including the posterior parietal and occipital regions [12]. Similarly, neuropsychological findings identify deficits across multiple domains of cognitive functioning, including set shifting, response inhibition, memory, and attention [13-17].

Most of the imaging literature to date is in adult rather than pediatric OCD. This is especially the case for studies that have used diffusion tensor imaging (DTI) to identify changes in brain white matter (WM) [18]. DTI is a magnetic resonance method that estimates the magnitude and direction of water diffusion, which is dependent on the underlying structure of brain tissue. In particular, highly organized myelinated axon fibers will constrain and direct water diffusion, such that DTI parameters allow inferences about WM organization and integrity. Mean diffusivity (MD) is the DTI parameter that reflects *magnitude* of diffusion at each voxel, which varies with tissue density regardless of fiber orientation [19]. Fractional anisotropy (FA) is the DTI parameter reflecting *directionality* of diffusion in each voxel, such that FA is higher along fiber bundles that are more coherent or organized [20]. FA can also be separated into components reflecting diffusion parallel and perpendicular to the WM tracts, referred to as the axial diffusivity (AD) and radial diffusivity (RD), respectively. FA values decrease when AD decreases and/or RD increases. Interestingly, RD and AD may be biomarkers of different cellular developmental or pathological processes. Specifically, changes in RD appear to be associated with cell membrane alterations (myelination), whereas variations in AD may be more related to axonal injuries (volume and organization) [21].

In DTI studies of adult OCD, FA has been the most commonly studied diffusion parameter. FA alterations have been observed most consistently in the corpus callosum (CC), cingulum bundle, internal capsule, and anterior thalamic radiation, as well as the parietal cortex (e.g., [22-25]). However, the directionality of FA alterations has varied, with reports of lower [23,24,26,27], higher [25,28], or comparable FA values when comparing adult OCD patients with healthy control (HC) subjects [22,29,30]. These seemingly disparate findings likely reflect true regional brain variability in hypo- and hyper-connectivity across fiber tracts implicated in OCD, as well as methodological differences across studies. For instance, although all studies have reported on FA, fewer have incorporated other diffusivity measures that can help contextualize FA findings. In addition, findings may relate to certain patient variables, such as psychiatric comorbidity, illness duration, and medication use. Notably, investigation of childhood OCD can help reduce some of these confounding influences including those related to illness chronicity [6] and can also isolate neurobehavioral features that relate to an early age at onset. Most adult OCD studies have combined patients with childhood and adult onset, which may introduce neurobiological variability. For instance, whereas meta-analyses of functional imaging findings indicate a central role of the caudate nucleus in adult OCD, neuroimaging studies of pediatric OCD point to more prominent involvement of other basal ganglia structures and the thalamus [5]. Thus, age at onset may be associated with neurobiological variability in OCD and could help parse disease heterogeneity.

As in the adult literature, studies of pediatric OCD have provided evidence of diffusion abnormalities in multiple WM tracts, although the nature and directionality of diffusion changes have varied, as have their associations with clinical features of illness. Two pediatric OCD DTI studies have reported FA as the main measure of diffusion [16,31]. Zarei et al. [31] found increased FA across a number of WM tracts in adolescent OCD patients, changes more widespread than those found in many adult OCD studies. Moreover, OCD symptom severity was positively correlated with FA in several of these tracts. Similarly, Gruner and colleagues [16] found that children with OCD had FA increases, though these were localized to four WM tracts, namely the left dorsal cingulum bundle, splenium of the CC, right corticospinal tract, and left inferior fronto-occipital fasciculus. Interestingly, increased FA in the cingulum bundle predicted better executive functioning within the OCD group, suggesting that it may reflect a compensatory process. Another two pediatric OCD studies found no group differences in FA, but examination of AD and RD revealed significant diffusivity differences [32,33]. Specifically, Silk et al. [33] found lower AD in the genu and splenium of the CC of children with OCD compared with controls, and lower AD correlated with greater severity of symptoms. In contrast, Jayarajan et al. [32] found significantly higher AD and RD, and neither was significantly correlated with symptom severity, medication dosage, or treatment duration. No pediatric OCD study has yet reported on whether diffusion changes are significantly associated with age at onset.

In this study, we used DTI to compare brain WM microstructure in youth with OCD compared with healthy matched controls, examining four diffusion parameters (MD, FA, AD, and RD). We hypothesized that pediatric OCD would be associated with altered FA, without an *à priori* prediction concerning the directionality of alteration (i.e., increased or decreased) due to scant published data in pediatric samples and inconsistent adult OCD DTI findings. Based on theoretical and empirical indications that age at onset may be relevant to the variability of DTI findings in OCD, we also tested the hypothesis that diffusion differences would be associated with age at onset of OCD.

## Methods

### Subjects

Thirty-six youth were enrolled in this neuroimaging experiment as paid volunteers. OCD subjects were treatment-seeking children presenting to an OCD clinic, and control subjects were recruited in the surrounding Boston metropolitan community via advertisements. The subjects were selected to be between 10 and 19 years old, similar to the age range in prior imaging studies of pediatric OCD. Participants were excluded if they reported current medical or neurological illness, or a history of head injury with loss of consciousness. Prior to enrollment, written informed consent was obtained from a parent/legal guardian and written informed assent was obtained from the child/adolescent participant. All study procedures were performed in accordance with the Human Research Committees at McLean Hospital and Partners Healthcare System.

The Kiddie Schedule for Affective Disorders and Schizophrenia (KSADS) was administered to all participants and their parents by doctoral-level psychologists [34]. Individuals included in the OCD group met DSM-IV criteria for this disorder based on the KSADS [1]. In addition, OCD symptom severity scores were determined using the Children’s Yale-Brown Obsessive-Compulsive Scale (CY-BOCS) [35]. To recruit a representative and generalizable OCD sample, the inclusion criteria for the OCD group allowed for comorbid psychiatric disorders, with the exception of psychotic disorders, bipolar disorder, mental retardation, substance use disorders, and pervasive developmental disorders. Neuroleptic and anti-hypertensive medications were exclusionary. All individuals included in the HC group were free from any current Axis I psychiatric disorder and psychotropic medications. All subjects completed the Child Depression Inventory (CDI) [36], and the Yale Global Tic Severity Scale (YGTSS) was used to rule out subjects with Tourette’s syndrome and other tic-related disorders [37].

The participants consisted of 17 children with OCD and 19 HC youth. This sample was obtained after excluding data from participants for excessive head movement (1 OCD) and poor head coverage (1 HC). The patient group endorsed the following types of OCD symptoms across previously identified clusters [38]: contamination/washing (*N* = 4); symmetry/arranging/counting/repeating (*N* = 10); and aggression, sexual, religious, and/or somatic obsessions/checking (*N* = 14). No hoarding symptoms were reported. Nine (9) of the patients had no comorbid Axis I diagnosis. The following comorbidities were present in the remaining eight OCD patients, based on KSADS interviews: generalized anxiety disorder (*N* = 1), specific phobia (*N* = 2), agoraphobia (*N* = 1), major depressive disorder (*N* = 2), depression not otherwise specified (*N* = 2), oppositional defiant disorder (*N* = 1), and attention-deficit hyperactivity disorder (*N* = 2). Three (3) of the OCD patients were not taking any psychotropic medication. Primary medications taken by the remaining 14 OCD subjects were antidepressants: selective serotonin reuptake inhibitors (*N* = 13) and tricyclic agents (*N* = 1). In addition, some patients were taking secondary medications, namely stimulants (*N* = 4), mood stabilizers (*N* = 3), and benzodiazepines (*N* = 1).

### Diffusion tensor imaging

#### Image acquisition

DTI scans were acquired using a Siemens Tim Trio 3T scanner at the McLean Hospital Imaging Center. Diffusion weighted imaging data were obtained in 60 directions with the following parameters: echo time = 98 ms, bandwidth = 1,396 Hz/pixel, matrix = 128 mm × 128 mm, FOV = 256 mm × 256 mm, NEX = 1, voxel size = 2.0 mm^3^ × 2.0 mm^3^ × 2.0 mm^3^, 10 T2 low *b* (*b* = 0 s/mm^2^), and 60 DWI (diffusion sensitivity *b* = 700 s/mm^2^), and 60 axial slices with 2-mm thickness.

#### Image processing and analysis

The analysis of DTI data was done using the FMRIB Diffusion Toolbox from the FSL processing software package (http://www.fmrib.ox.ac.uk/fsl) [39,40]. The first step was motion and eddy current distortion correction, applied using FSL’s *eddy_correct* tool, which ran with its default options. The raw data were skull-stripped using FSL’s Brain Extraction Tool (BET) [41]. A diffusion tensor model was fit at each voxel using a least squares fit to the diffusion signal with FSL’s *dtifit* tool; this generated maps for each of the diffusivity measures (FA, MD, AD, and RD). At this point, a mathematical correction for systematic vibration artifact was applied [42]. Voxel-wise processing of diffusivity measures was carried out using tract-based spatial statistics (TBSS) [43], which is part of FSL [44]. Images from all subjects were aligned to each other using nonlinear registration in order to determine the most representative individual (i.e., the closest to the mean of the group) to be defined as the target image. This target image was then aligned, using affine registration, to MNI152 standard space. Each individual subject was then registered into the Montreal Neurological Institute (MNI) space by combining the nonlinear transform (generated via FSL’s FNIRT) from the subject to the target image with the affine transform from the target image into the MNI space. A mean FA image was created by averaging all aligned FA maps and was thresholded with an FA ≥ 0.2 to generate a mean FA skeleton, which represents the centers of all fiber tracts common to all subjects. Each subject’s aligned FA image was projected onto the mean FA skeleton and served as the input to the TBSS. Group statistical analysis was then conducted only on voxels within the white matter skeleton mask, therefore restricting the voxel-wise analysis only to voxels with high confidence of lying within equivalent major white matter pathways in each individual. After completing the above procedures for FA, the nonlinear warps and skeleton projection were applied to MD, RD, and AD using tbss_non_FA. Differences in FA, MD, and axial and radial diffusivity between the OCD and control groups were assessed using voxel-wise independent two-sample *t*-tests by randomization, the nonparametric analysis tool in FSL. The Threshold-Free Cluster Enhancement (TFCE [45]) option was employed at family-wise error-corrected *p* < 0.05 to obtain cluster inferences.

### Statistical analyses

Group differences in demographic characteristics were examined using *χ*^2^ tests for categorical variables and independent *t*-tests for continuous variables. DTI parameters (FA, RD, MD, and AD) were analyzed using permutation testing and TFCE in FSL by applying an independent *t*-test to the data for between-group comparisons. The significance level was *p <* 0.05, family-wise error-corrected. Within significant clusters, the mean values were computed. Correlations of DTI variables with demographic and clinical variables were performed in SPSS version 20.

## Results

Demographic and clinical data are summarized in Table 1. CY-BOCS total scores ranged from 8 to 32, spanning the mild to extreme severity range of obsessive and compulsive symptoms. In addition, OCD patients endorsed significantly higher levels of depression than HC subjects on the CDI (*p*’s < 0.01).

**Table 1:** **Demographic and clinical characteristics of the sample (mean ± SD or** ***N*** **(%))**

**Variable**	**OCD (** ***n*** **= 17)**	**Controls (** ***n*** **= 19)**	***p***
Female	6 (35%)	6 (32%)	0.8
Age (years)	14.06 ± 2.56	13.58 ± 2.12	0.54
Education (years)	8.65 ± 2.40	8.37 ± 1.95	0.70
CDI (total)	8.94 ± 7.16	3.47 ± 3.79	<0.01
CY-BOCS (total)	17.06 ± 8.17	-	
Obsessions	8.12 ± 4.47	-	
Compulsions	8.94 ± 4.19	-	
Age at onset (years)	8.82 ± 3.36	-	
Duration of illness (years)	5.24 ± 2.97	-	

### Between-group differences in diffusion

**Figure 1:**
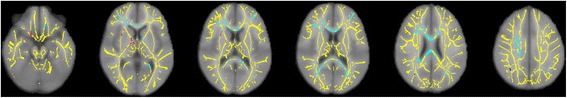
**Clusters of significantly lower fractional anisotropy in youth with obsessive-compulsive disorder compared with healthy controls (skeleton: yellow; cluster 1: light blue; cluster 2: red; cluster 3: dark blue; cluster 4: bright green; cluster 5: copper; cluster 6: magenta; cluster 7: light green).**

**Figure 2:**
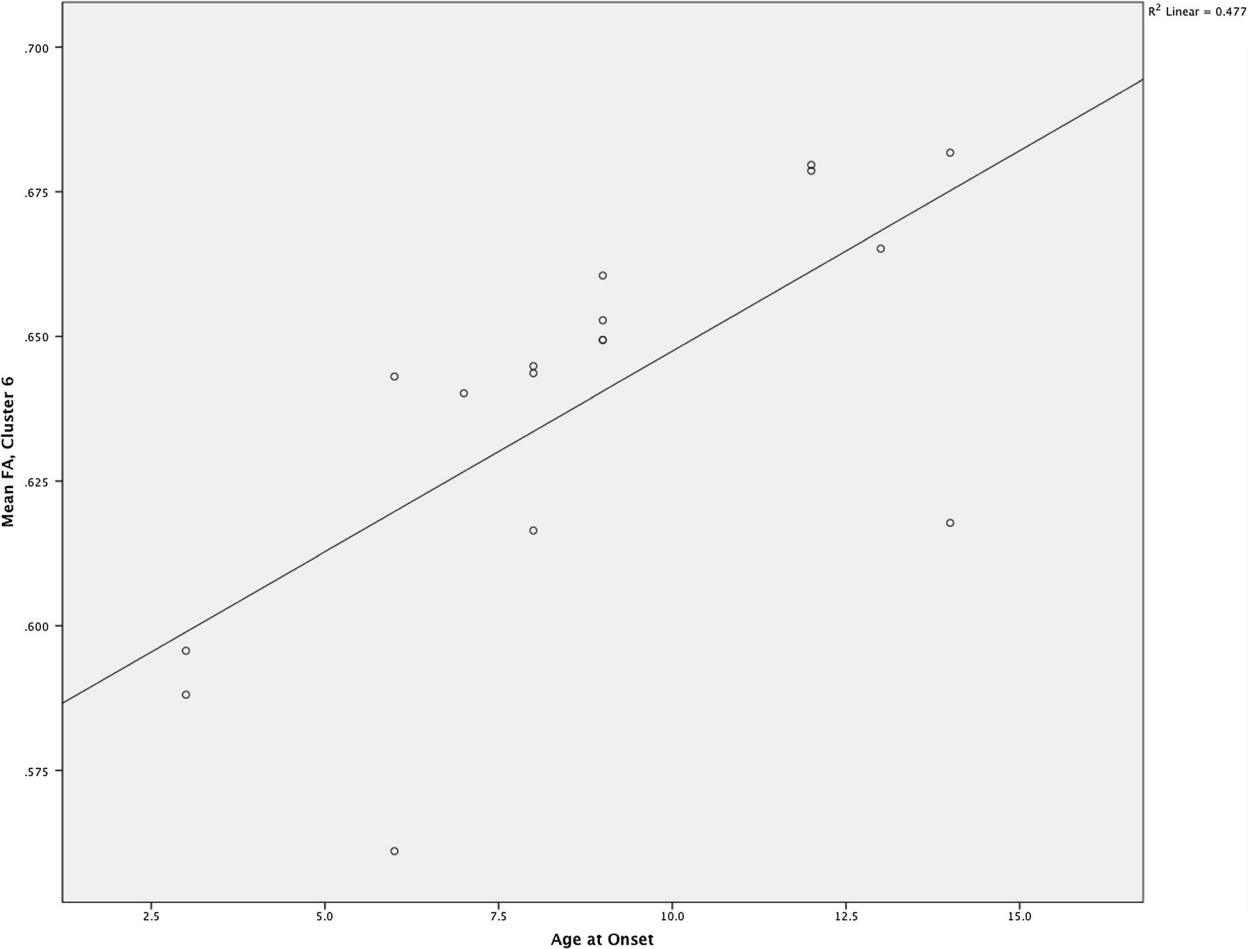
**Association of age at onset with fractional anisotropy (FA) in youth with OCD in cluster 6.**

**Figure 3:**
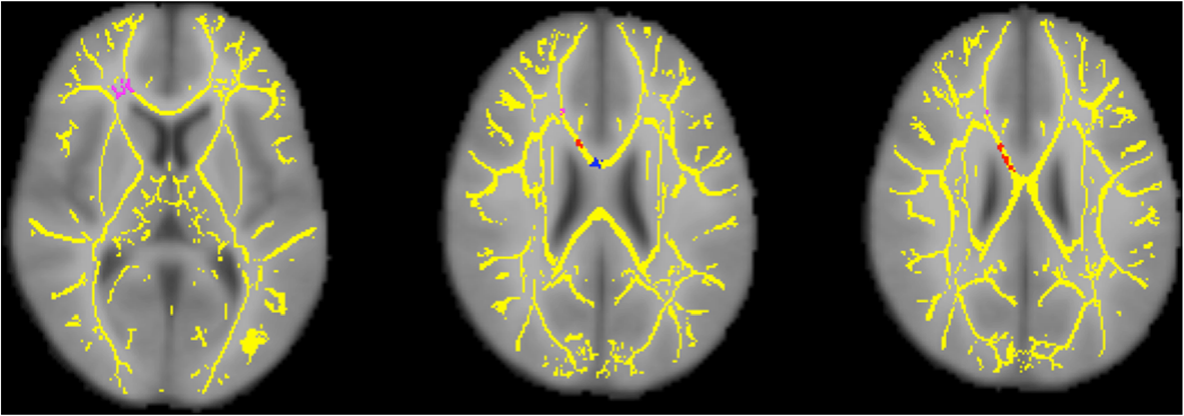
**Clusters of significantly higher radial diffusivity in youth with obsessive-compulsive disorder compared with healthy controls (skeleton: yellow; cluster 1: magenta; cluster 2: red; cluster 3: blue).**

OCD youth had significantly lower FA than HC subjects in widespread areas across seven separate clusters, numbered 1–7 in Table 2 and Figures 1 and 2. Nearly 98% of the significant voxels localized to clusters 1 and 2. Cluster 1 was a bilateral cluster (9,022 voxels) encompassing areas of the frontal lobes and CC (genu, body, and splenium). Cluster 2 (1,454 voxels) encompassed areas of the anterior cingulate cortex and extended into several subcortical regions including the putamen, amygdala, and thalamus. Cluster 3 (132 voxels) localized to the right angular and lateral occipital gyri. Cluster 4 (50 voxels) was in the right inferior frontal cortex, more specifically the subcallosal cortex. Cluster 5 was also a right inferior frontal cluster but more anteriorly, corresponding to the orbitofrontal cortex. Cluster 6 corresponded to 14 voxels in the right thalamus, and cluster 7 comprised 6 voxels in the right caudate and anterior internal capsule. There were no significant clusters where FA was higher in OCD patients than in HC subjects. Compared with HC subjects, OCD patients also had significantly increased RD in areas of the right frontal cortex and body of the CC, across three clusters that overlapped with the first FA cluster (Table 3; Figure 3). There were no significant clusters where RD was lower in OCD patients than in HC subjects. There were no statistically significant group differences in MD or AD.

**Table 2:** **White matter clusters with reduced fractional anisotropy (FA) in pediatric OCD patients versus healthy controls**

**Cluster label and anatomical localization**	**Voxels**	**MNI coordinates**			**FA**		
		***x***	***y***	***z***	**OCD**	**HC**	***p***
1. Bilateral frontal (ATR, UF, IFOF, forceps minor, anterior corona radiata) and corpus callosum (genu, body, splenium)	9,022	20	37	6	0.533 ± 0.191	0.573 ± 0.188	0.026
2. Right cingulate and basal ganglia: anterior cingulate cortex (UF), anterior limb of IC (ATR), putamen (UF), amygdala (ILF), cerebral peduncle (CST), thalamus, and posterior limb of IC (ATR)	1,454	23	0	−9	0.419 ± 0.157	0.460 ± 0.158	0.035
3. Right posterior cerebral cortex: lateral occipital and angular gyri (SLF)	132	36	−56	33	0.423 ± 0.100	0.500 ± 0.098	0.048
4. Right inferior frontal lobe (subcallosal cortex)	50	8	21	−19	0.296 ± 0.067	0.370 ± 0.107	0.049
5. Right inferior frontal: orbitofrontal cortex	36	8	37	−22	0.267 ± 0.067	0.323 ± 0.073	0.050
6. Right thalamus	14	18	−5	11	0.639 ± 0.041	0.668 ± 0.038	0.050
7. Right caudate, anterior IC (ATR)	6	22	−4	24	0.430 ± 0.056	0.493 ± 0.081	0.050

*ATR* anterior thalamic radiation, *CST* corticospinal tract, *IC* internal capsule, *IFOF* inferior fronto-occipital fasciculus, *ILF* inferior longitudinal fasciculus, *SLF* superior longitudinal fasciculus, *UF* uncinate fasciculus.

**Table 3:** **White matter clusters with increased radial diffusivity (RD) in pediatric OCD patients versus healthy controls**

**Cluster label and anatomical localization**	**Voxels**	**MNI coordinates**			**RD**		
		***x***	***y***	***z***	**OCD**	**HC**	***p***
1. Right frontal (anterior corona radiata, forceps minor, ATR; overlaps FA cluster 1)	406	19	32	12	0.000655 ± 0.000085	0.000594 ± 0.000089	0.042
2. Right body of the corpus callosum (overlaps FA cluster 1)	81	12	4	28	0.000421 ± 0.000085	0.000358 ± 0.000083	0.049
3. Right body of the corpus callosum (close to midline; overlaps FA cluster 1)	49	3	4	23	0.000474 ± 0.000084	0.000399 ± 0.000077	0.049

*ATR* anterior thalamic radiation.

*Post hoc* analyses showed that the mean FA and RD of clusters that differed between the diagnostic groups did not differ significantly between OCD patients with and without current comorbid disorders.

### Clinical correlates of diffusion changes

#### Age at onset

To examine relationships with age at onset, the mean FA and mean RD were extracted from the seven FA clusters and the three RD clusters characterized above. Lower age at onset was associated with significantly decreased FA in FA cluster 6 (right thalamus: Figure 2), *r*(15) = 0.691, *p* = 0.002, and with significantly increased RD in RD cluster 2 (right body of the CC), *r*(15) = −0.552, *p* = 0.022. The correlation between age at onset and FA in the thalamus remained significant after partialing out age, *r*(14) = 0.636, *p* = 0.008, though the correlation between age at onset and RD in the right body of the CC was reduced to a trend level after partialing out age, *r*(14) = −0.435, *p* = 0.093. After partialing out duration of illness, the correlations between both DTI variables and age at onset remained significant: for the FA cluster, *r*(14) = 0.556 and *p* = 0.022; for the RD cluster, *r*(14) = −0.520 and *p* = 0.039.

#### Symptom severity

The mean FA and mean RD from the identified clusters were also examined in relation to symptom severity. There were no significant correlations with CY-BOCS total scores, CY-BOCS compulsive symptom scores, or CY-BOCS obsessive symptom scores. CY-BOCS total scores were not significantly correlated with age, age at onset, or duration of illness.

## Discussion

These findings add to emerging evidence of abnormal integrity of brain WM tracts in pediatric OCD. This is the first DTI study to report that children with OCD compared with healthy youth show regional brain *reductions* in FA. Over 84% of the significant voxels localized to a single cluster that encompassed a large expanse of bilateral frontal cortex and extended into the CC. Smaller clusters of FA reduction were seen in the right posterior parietal and occipital cortices and the subcallosal and orbitofrontal cortices, as well as the thalamus and putamen. Youth with OCD also had significantly increased RD in areas that overlapped with the largest cluster of FA reduction, including the right anterior cingulate cortex and right body of the CC, suggesting deficient myelination in these areas. Finally, an earlier onset of OCD was associated with more pronounced FA reductions in the right thalamus and greater RD increases in the right body of the CC. Overall, our results are in line with increasing evidence that brain WM alterations are present in pediatric OCD and support a possible moderating role of age at onset on some aspects of OCD pathophysiology.

We found prominent alterations of frontal WM in subjects with OCD, affecting fiber tracts that connect the frontal lobe to both cortical and subcortical regions. Thus, OCD youth exhibited lower FA in a large cluster of bilateral frontal cortex, including anterior cingulate cortex (BA 32) and orbital-frontal cortex (BA 11), areas that are considered central to OCD pathophysiology [4,7]. This cluster overlapped with multiple WM tracts, including projection and association fibers that extend to the thalamus, anterior temporal-limbic regions, and parietal and occipital cortices. Consistent with frontal-subcortical models of OCD, FA reductions were also seen in the putamen, caudate, and thalamus of OCD youth. In adult DTI studies, both increased and decreased frontal FA have been found in OCD (e.g., [22,24]) and this may relate in part to illness heterogeneity. One source of variance in DTI studies of adult OCD appears to be the relative predominance of genetic versus environmental etiologic factors [29]. In their adult sample of monozygotic twins concordant and discordant for OCD, den Braber et al. [29] found that some WM tracts (including frontal tracts) show FA alterations that are in the opposite direction in subjects at high genetic risk compared with subjects at high environmental risk for OCD. Although neither the current nor prior DTI studies of pediatric OCD formally assessed family history, it is possible that our sample was relatively high on genetic loading given its particularly early age at onset (averaging several years earlier than in prior studies [31,32]) and that this would explain findings of reduced FA in our sample but not prior pediatric OCD studies. Future DTI studies could be designed to parse the influences of genetic and environmental influences on WM in pediatric OCD.

Consistent with a growing body of neurobehavioral evidence that posterior association cortices are implicated in OCD, we found reduced WM integrity in the angular and lateral occipital gyri. A number of functional imaging studies have found abnormalities of glucose metabolism, cerebral blood flow, and brain activation in the posterior cortices of adult OCD patients [12]. Neuropsychological studies have shown that adults with OCD are impaired on visuospatial and decision-making functions that rely on the integrity of the parietal lobe [46-48]. In addition, a magnetic resonance spectroscopy study found increased choline in the parietal lobe WM of OCD, indicative of increased phospholipid turnover of myelinated axons in this region [49]. Although prior childhood OCD DTI studies found evidence of CC alterations suggestive of posterior association cortex involvement, the current study is the first to identify a cluster of altered FA in parietal cortex of pediatric patients. In DTI studies of adult OCD, reduced FA in parietal WM also has been found to distinguish patients from healthy control subjects (e.g., [12,24]). Thus, our results extend those of adult DTI studies by indicating that reduced parietal WM integrity is present in pediatric patients. This finding converges with a report of reduced parietal WM volume in adolescents with OCD compared with healthy youth [50]. Neurodevelopmental studies have shown that increases in FA and other maturational changes occur in parietal-occipital WM structure from the earliest years of childhood [51], such that disruption of this process could relate to early-onset pathophysiology [52]. In adult studies, visuospatial deficits and intrusive visual imagery are prominent features of OCD and have been documented premorbidly in adults prior to their onset of OCD symptoms [53]. Altogether, this suggests that abnormal parietal-occipital lobe structure and its behavioral manifestations are present in childhood OCD and early in the course of adult OCD.

Findings of diffusion alterations in the CC add to growing evidence of interhemispheric abnormalities in OCD, evident in both the pediatric and adult literatures [18,54]. In this study, OCD compared with healthy youth had reduced FA in portions of the genu, body, and splenium, which implicates fibers that connect bilateral frontal, parietal, and temporal-occipital association cortices [55,56]. There was a prominent cluster of FA reduction in the middle to posterior body of the CC, which connects bilateral somatosensory cortices and posterior parietal regions [57]. We also found decreased FA in the anterior portion of the genu, where small axons connect bilateral prefrontal cortex and ventral prefrontal cortex to the striatum. In an earlier study of pediatric OCD, Zarei et al. [31] had found increased FA in somewhat different CC areas corresponding to the posterior genu and anterior body of the CC, where larger axons connect primary motor cortices. Thus, it is possible that directional coherence of fiber tracts is differentially altered across different sections of the genu and body of the CC in childhood OCD, indicating variable pathology across topographically distinct association cortices.

Our finding of decreased splenium FA conflicts with two prior reports on youth with OCD [16,31]. Both investigations found higher splenium FA, which correlated with significantly greater OCD symptom severity [16,31]. Both of these prior studies had a lower proportion of medicated OCD patients (61%, 52%) than the current study (82%). Moreover, significantly elevated splenium FA was seen only among the subgroup of unmedicated patients in one of those studies [16]. Thus, it may be that our finding of lower splenium FA reflects the effects of medication treatment. Our study design does not allow us to test this question, but there is some prior evidence that treatment-naïve OCD patients, including pediatric patients, have higher FA, greater WM density, and larger size of the CC [57]. Moreover, in a longitudinal study of adults with OCD, Yoo et al. [25] found that drug-naïve patients had multiple areas of increased FA (including in the posterior CC) and that these normalized after a course of clinically effective citalopram treatment. Another possible source of conflicting findings across DTI studies is heterogeneity of OCD clinical features (e.g., our sample included a minority of “washers” relative to “checkers”). The adult OCD literature has begun to explore neural correlates of symptom dimensions in OCD [58]. Similar studies could be designed in pediatric OCD by selecting more homogeneous patient samples.

Alterations in diffusion anisotropy (FA) can result from changes in either RD (perpendicular) and/or AD (parallel), and these subcomponents are differentially modulated by myelin and axonal degeneration mechanisms, respectively [59]. In this study, we found concomitant decreases in FA and increases in RD in the right frontal cortex and right body of the CC, a pattern thought to reflect deficient myelination [59-62]. Abnormal myelination also has been implicated in OCD by other lines of research, including magnetic resonance spectroscopy findings of increased levels of cell membrane breakdown products in OCD youth [63,64], and genetic evidence of an association between OCD and a gene (OLIG2) involved in the development of oligodendrocytes [65]. Similar to our study, Jayarajan and colleagues [32] also had found significantly increased RD in a DTI in children with OCD compared with controls, although FA did not differ significantly between groups. Because increased RD was accompanied by increased AD in their patient sample, Jayarajan et al. [32] interpreted this as indicative of hyperconnected yet insufficiently myelinated WM tracts in affected regions. In contrast, Gruner et al. [16] found decreased RD in the context of significantly increased FA in four WM regions of pediatric OCD compared with control subjects, pointing to excessive myelination of certain axon fibers. Finally, Silk et al. [33] found significantly decreased AD in the genu and splenium of the CC of OCD youth, which could indicate less coherently organized callosal axons; FA was not significantly different between the groups in the CC or any other WM tract of that study. Thus, in the relatively small literature to date, there are different patterns of FA/RD/AD findings across pediatric OCD DTI studies. This could indicate involvement of multiple possible combinations of WM microstructure alterations in pediatric OCD wherein both myelin and axonal changes may be present in the context of either reduced or increased coherence of fiber tracts. The pattern of abnormalities may vary across brain regions, due to age-related maturational processes [16,31] and/or as a function of clinical phenomenology. These questions will need to be disentangled by conducting additional studies in this relatively young area of study.

Our findings suggest that age at onset may be an influential moderating factor on some of the WM changes in pediatric OCD. On average, our patients had an earlier onset of illness (average 8 years) than those in the two earlier pediatric OCD studies that reported on age at onset (11 years [31] and 13 years [32] on average). Moreover, within our patient sample, earlier onset was associated with significantly lower FA in the right thalamus and significantly higher RD in the right CC. The former association remained significant even after controlling for age, suggesting that earlier onset of OCD disease-related processes is associated with reduced integrity of WM in the thalamus. Involvement of the thalamus in early-onset OCD is consistent with the conclusions of a literature review that prominent thalamic abnormalities may be a component of a slightly different neuropathological substrate of childhood-onset versus adult-onset OCD [54].

Studying pediatric patients near the onset of their illness helps identify neurobiological changes that may be more primary than those arising later on in the course of illness, although developmental and disease effects may still be influential. Brain alterations noted during childhood and adolescence may change in their nature due to ongoing maturation of both gray matter and WM, which may contribute to variable findings across studies. In addition, compulsively engaging in a particular type of behavior or cognitive process can change brain structure [4,66,67]. Thus, even in pediatric OCD, anatomical brain differences may reflect a consequence rather than a precursor of the disorder. In the only study of neuropsychological correlates of DTI measures in pediatric OCD, the pattern of results found by Gruner et al. [16] suggested that certain FA increases might reflect compensatory increases in WM coherence to mitigate neuropsychological deficits. This highlights the usefulness of studying patients with OCD as close to symptom onset as possible, when such epiphenomenal changes may be less prominent.

Our findings should be interpreted in the context of the study’s limitations. This was a cross-sectional investigation, and our results are therefore correlational in nature. We cannot rule out that these findings were affected by comorbidity within our OCD sample, particularly depression and other anxiety disorders present in a third of the patients. However, in *post hoc* analyses, severity of depression and anxiety symptoms was not associated with FA or RD in any of the significant clusters. Moreover, only a small percentage of the sample had any particular comorbid condition, making it implausible that the group findings were driven by a particular comorbidity. Similarly, we cannot exclude the possibility of medication effects, since most of our patients were medicated; although none of the previous pediatric OCD studies found any indication that DTI parameters were affected by medication use [16,31,32], it is possible that this reflected limited power to detect such effects. Finally, this and other pediatric OCD studies to date have not had sufficient power to examine the relationship of DTI WM measures with OCD symptom profile, which have been found to differentially relate to regional brain abnormalities in adult OCD [68].

## Conclusions

Our findings of significantly lower FA and higher RD in childhood OCD are consistent with compromised WM integrity and reduced myelination, particularly involving the CC and fiber tracts that connect the frontal lobes to widespread cortical and subcortical targets. Our findings also suggest that age at onset may be a moderator of some of the WM changes in children with OCD. DTI research in childhood OCD is still in its infancy, and future studies should incorporate examination of how genetic and environmental risk factors, as well as aspects of illness phenomenology, may help parse divergent findings across studies.

## Abbreviations

AD: Axial diffusivity
CC: Corpus callosum
CDI: Child Depression Inventory
CY-BOCS: Children’s Yale-Brown Obsessive-Compulsive Scale
DTI: Diffusion tensor imaging
FA: Fractional anisotropy
HC: Healthy control
KSADS: Kiddie Schedule for Affective Disorders and Schizophrenia
MD: Mean diffusivity
RD: Radial diffusivity
OCD: Obsessive-compulsive disorder
TBSS: Tract-based spatial statistics
TFCE: Threshold-Free Cluster Enhancement
WM: White matter
YGTSS: Yale Global Tic Severity Scale
